# Emergency Department Evaluation and Care of Hepatic Encephalopathy: ACEP Expert Panel Recommendations

**DOI:** 10.5811/westjem.63064

**Published:** 2026-08-17

**Authors:** Christopher W. Baugh, Arun B. Jesudian, Stanley C. Thompson, Christi Jen, William Ford, James F. Neuenschwander, Edgardo Ordonez, Alpesh N. Amin

**Affiliations:** * Brigham and Women’s Hospital, Department of Emergency Medicine, Boston, Massachusetts; † Weill Cornell Medicine, Division of Gastroenterology and Hepatology, New York, New York; ‡ TeamHealth Lifepoint Group, Nashville, Tennessee; § Mayo Clinic, Department of Pharmacy, Phoenix, Arizona; ¶ Baylor College of Medicine, Henry J. N. Taub Department of Emergency Medicine, Houston, Texas; || Jefferson Health, Division of Hospital Medicine, Abington, Pennsylvania; # Ohio State University, Department of Emergency Medicine, Columbus, Ohio; ** University of California, Irvine, Department of Medicine, Irvine, California

## Abstract

**Introduction:**

Patients with hepatic encephalopathy frequently visit the emergency department, and these visits are expected to rise in the coming years due to an increased prevalence of liver disease in the United States. Accordingly, we sought to define a comprehensive and efficient approach for emergency physicians to evaluate, treat, and determine the optimal disposition of patients presenting with possible or confirmed hepatic encephalopathy and its complications.

**Methods:**

We reached consensus recommendations through a structured literature review and a modified Delphi technique, informing an expert panel of academic and community emergency physicians convened by the American College of Emergency Physicians.

**Results:**

We created the assess, look, treat, evaluate risk, reassess, disposition (ALTERD) framework as a digital point-of-care tool to support clinicians in guiding key bedside steps involved in the diagnosis and care of patients across the spectrum of hepatic encephalopathy.

**Conclusion:**

A collaborative expert panel process can create an emergency department-focused, easily accessible, and comprehensive digital tool to complement workflows and improve the care of a vulnerable and growing patient population.

## INTRODUCTION

Chronic liver disease affects approximately 2.6% of the population in the United States (U.S.), with prevalence expected to rise.^1^,^2^ Metabolic dysfunction-associated steatotic liver disease increased from 20.0% (1988–1994) to 31.9% (2013–2016).^3^ While recent breakthroughs in hepatitis C treatment have been introduced, the ongoing opioid crisis associated with injection drug use has allowed it to remain a leading cause of liver disease in the U.S., with over 67,000 new cases in 2022.^4^ Physicians are increasingly likely to encounter patients with chronic liver disease. This trend underscores the need for effective screening tools to prompt further evaluation, such as the serum-based fibrosis-4 (FIB-4) index.^5^ Hepatic encephalopathy (HE) is a severe cirrhosis complication affecting approximately 202,000 U.S. adults, with high recurrence risk, frequent readmissions, and poor survival.^6^,^7^ Hepatic encephalopathy is characterized predominantly by alterations in personality, consciousness, cognition, and motor function. Its manifestation may not be clinically obvious, and multiple detection tools contribute to variation in reported incidence and prevalence.^8^ Impaired hepatic ammonia metabolism and portal hypertension shunt ammonia-rich portal blood into the systemic circulation, where it crosses the blood-brain barrier and is converted to glutamine, causing cerebral dysfunction.^9^

Given the current and anticipated burden of liver disease in the U.S., efforts should be focused on more effective identification and treatment in key settings, such as the emergency department (ED). Overt HE occurs in 30–40% of cirrhosis patients during their clinical course.^10^ Hepatic encephalopathy is broadly classified as either minimal or covert (ie, with normal mental status and neurologic exam but with psychometric testing abnormalities detected on tests such as the Psychometric Hepatic Encephalopathy Score, which is out of scope for most ED visits) or overt (ie, with neurologic and neuropsychiatric abnormalities).^11^,^12^ The West Haven criteria (WHC) for the diagnosis of HE is found in the American College of Emergency Physicians (ACEP) HE point-of-care tool, which grades HE from subtle cognitive impairment to coma (Table 1).^13^ Minimal HE or covert HE occurs in 20%–80% of patients with cirrhosis.^14^,^15^ Overt episodes of HE are debilitating, can occur without warning, render the patient incapable of self-care, and frequently result in hospitalization. The likelihood of developing HE correlates with the severity of liver disease.^10^

**Population Health Research Capsule**

What do we already know about this issue?

*Hepatic encephalopathy is a common, high-risk emergency department (ED) presentation, but diagnosis and management vary, and ED-specific guidance has been limited*.

What was the research question?

*How should ED clinicians evaluate, treat, and determine disposition for hepatic encephalopathy?*

What was the major finding of the study?

*An expert panel developed the ALTERD bedside framework for ED hepatic encephalopathy care*.

How does this improve population health?

*A standardized ED tool supports earlier recognition, consistent care, appropriate disposition, and improved transitions for patients with hepatic encephalopathy*.

Distinguishing HE from other conditions in the ED setting can be challenging, leading to underdiagnosis. Common differential diagnoses include medication adverse effects, electrolyte disorders (hypoglycemia, hyponatremia, hypercalcemia), uremia, sepsis, central nervous system infection, nonconvulsive epilepsy, psychiatric disorders, alcohol intoxication or withdrawal, hypercapnia, and intracranial bleeding or stroke.^16^ Undertreatment contributes to recurrence, despite evidence demonstrating that the rifaximin with or without lactulose regimen is cost-effective approximately 99% of the time for preventing recurrence.^17^,^18^ Given HE’s increasing prevalence and challenging clinical presentations, we developed a consensus-based bedside decision tool to help emergency physicians assess and manage patients with suspected or confirmed HE within typical ED workflows.

## METHODS

### Study Design and Setting

Between July and December 2021, the ACEP convened an expert panel on HE. We conducted a structured literature review, serving as a rapid evidence assessment that balanced the rigor of a systematic review with a more focused scope to guide and support the panel’s recommendations. We searched PubMed and included English-language sources with full text available using the keywords “hepatic encephalopathy.” Panel chairs reviewed abstracts with an emphasis on recency (eg, within five years), relevance to ED clinical care, journal impact factor, study design, and specialty society affiliations and guidelines. Additionally, they reviewed the references of highly relevant papers to identify and include foundational sources most closely related to the topic. The panel chairs circulated final full-length selections among the entire group for subsequent inclusion as supporting evidence for the proposed tool content. We employed a modified Delphi technique to achieve consensus via virtual panel meetings.^19^ Panel chairs assigned major sections to pairs of panelists, who drafted proposed content based on the literature review. In four rounds of subsequent voting across the entire panel, including comments with suggested edits, the content was iterated until a majority consensus of over 50% was reached.

### Selection of Participants

The panel consisted of a multidisciplinary group of clinicians from diverse geographical areas and practice settings. The ACEP staff selected the co-chairs (ABJ and SCT), who then selected the panelists. Selection criteria included direct clinical experience in evaluating and managing the target patient population in the ED setting, and the final panel consisted of three academic emergency physicians, two community emergency physicians, one ED pharmacist, one hepatologist, one case manager, and one hospitalist. Though an unrestricted educational grant from Salix Pharmaceuticals to ACEP offset project management expenses, including honorariums, there was no industry involvement in designing, developing, or editing the work product.

### Interventions

We conducted four rounds of structured voting; for each round, the chairs presented the initial voting results and facilitated extensive discussion during virtual meetings. The fourth round involved one additional survey of panelist comments and votes. The chairs modified the recommendations based on the discussion and voting from the previous rounds. The final panel recommendations represent consensus and majority opinions. This project relied on publicly available sources and was framed as a quality improvement project exempt from our institutional review board’s oversight.

## RESULTS

We developed the acronym ALTERD to communicate the six main steps for diagnosing ED patients with confirmed or suspected HE: assess, look, treat, evaluate risk, reassess, disposition. In Figure 1, we show the tool’s menu page, which features each of these steps along with supporting evidence and acknowledgments. Clicking each one reveals additional detail in bullet-point format, facilitating rapid referencing and usability. The ALTERD tool has been available since June 2023 on the ACEP website without a paywall. It is also available via the ACEP smartphone application on the Apple App Store or Google Play Store at no cost to current ACEP members in the “Point-of-Care” section. It is intended to be accessed in real time during an ED visit, providing clinicians with fast, helpful guidance. The website for this tool has had 412 visitors, and the application has had 355 users access it since its inception.

### Assess

The assessment begins with the chief complaint and initial history. Consider the diagnosis of HE in any patient presenting with altered mental status, particularly those with known liver disease. Due to acute delirium, obtain collateral information from family, caregivers, emergency medical services, or nursing home staff. Inquire about previous liver disease, alcohol use, recent procedures (eg, transjugular intrahepatic portosystemic shunt), nonadherence to lactulose/rifaximin, benzodiazepine or opioid use, diuretics, valproic acid, and infection symptoms. Investigate recent high-protein dietary loads and changes in bowel movements, including diarrhea, constipation, or bleeding.^16^

### Look

Diagnosing overt HE requires excluding alternative causes of altered mental status. Key physical exam findings in patients with liver disease are illustrated in Figure 2. The laboratory and diagnostic testing evaluation expected to be most helpful is illustrated in Table 2. While blood ammonia levels are commonly used, high levels alone add little diagnostic, staging, or prognostic value in HE patients with chronic liver disease.^8^ Repetitive ammonia measurements are neither predictive nor cost-effective. Although HE patients typically have elevated serum ammonia levels, the severity of HE does not correlate with these levels beyond a certain threshold.^20^

### Treatment

Table 3 correlates WHC scores with treatment regimens. Besides supportive care, identifying and treating precipitating factors is essential; nearly 90% of patients improve when these factors are corrected, thereby preventing recurrence and readmissions.^21^ Common precipitants include infections (eg, spontaneous bacterial peritonitis, urinary tract infections), gastrointestinal bleeding, electrolyte abnormalities, acute kidney injury, hypovolemia, sedating medications or intoxication, and portal vein thrombosis.^16^

Acute overt HE treatment includes reducing nitrogenous gut load via lactulose and rifaximin. Treatment goals are to induce remission by purging colonic bacterial contents and to maintain remission with secondary prophylaxis, given the high risk of recurrence. Lactulose, a nonabsorbable disaccharide, acidifies the gastrointestinal (GI) tract and inhibits ammonia production by coliform bacteria.^22^,^23^ Rifaximin, a semisynthetic nonsystemic antibiotic, decreases intestinal ammonia production and absorption by altering GI flora and is almost wholly excreted unchanged in feces. Studies show rifaximin equals or exceeds lactulose efficacy, with international guidelines supporting its use (GRADE 1, A, 1) as add-on therapy to lactulose for overt HE recurrence prevention.^24^–^27^ Polyethylene glycol (PEG) is an osmotic laxative that acts as a fecal cleanser, removing fecal nitrogen, and can be added in more severe cases.^28^ Additional treatments less commonly encountered in the ED but supported by evidence include intravenous L-ornithine L-aspartate (LOLA), oral branched-chain amino acids, and zinc.^24^

### Evaluate Risk

Patients may have dynamic exams during ED evaluation, requiring serial assessments. Emergency physicians should document appropriate WHC scores. Trends in mental status, vital signs, and laboratory values clarify patient trajectory and treatment response. Some patients with overt HE requiring hospitalization may be suitable for either floor or intensive care unit (ICU) admission. Hard ICU indications typically include intubation with mechanical ventilation, vasopressor support, frequent blood sugar and other electrolyte monitoring, and similar interventions deemed out of scope for inpatient floor care. Clear communication with consultants regarding their current status and decompensation potential is critical.

### Reassess

The broad diagnostic workup for overt HE typically spans hours and requires multiple reassessments. Diagnostic results refine the differential diagnosis and exclude alternative causes. This period allows reviewing vital sign trends, reassessing mental status, and detecting dynamic exam findings. For anticipated discharges, subsequent evaluations provide opportunities to screen for social determinants impeding outpatient treatment and follow-up. Engage case managers, social workers, or community health workers for vulnerable patients when available, or use asynchronous post-discharge referral systems during off-hours.

### Disposition

Once HE is confirmed, select the appropriate disposition and treatment that match the patient’s needs. In Figure 3, we provide suggested language for documentation that can easily be added to the medical decision-making section of a note to memorialize these actions. We correlate the range of disposition options to the severity of HE presentation in Table 3. Among patients with covert HE detected during an unrelated ED visit, they should be discharged home and scheduled for outpatient follow-up. If the patient does not have a primary care physician, local referral processes should be used. Patients with covert HE related to the ED chief complaint can also be discharged home, but with a specialty gastroenterology/hepatology referral. For discharged patients, see Figure 3 for an example handout to include with the after-visit summary. Consider an observation status hospitalization among patients with an established history of HE presenting with mild HE symptoms but without a safe discharge plan. Consider starting lactulose and rifaximin in the ED for treatment and prevention of recurrence, correcting metabolic disturbances, and a gastroenterology consult.

Patients with overt HE will require treatment with lactulose and rifaximin, followed by inpatient hospitalization on a hospital medicine service with a gastroenterology consult. These patients will have a WHC score of 2–3, indicating they will be arousable and redirectable, with minimal or moderate metabolic derangement or complications. Lastly, some patients with overt HE will require medical ICU hospitalization. Such patients will have a WHC score of 4 and will be obtunded and/or have severe metabolic disarray or complication. They will be unlikely to take medications by mouth, which may require alternative treatment strategies, such as rectal lactulose and/or extemporaneously compounded rifaximin or lactulose administered via gastric tube in addition to correction of metabolic abnormalities and resuscitation of other associated complications.^29^

A gastroenterology consult in the ED would be helpful for this population, along with other specialists as needed. Finally, regardless of patient disposition, engaging case management to ascertain prescription drug benefit coverage for rifaximin and/or patient assistance programs will prevent delays in initiation and gaps in treatment and, in turn, increase treatment retention to avoid rehospitalizations and other adverse outcomes associated with recurrent HE.^30^

## DISCUSSION

Standardized approaches to HE diagnosis and management are crucial for improving efficiency, achieving better outcomes, and ensuring appropriate ED care transitions. Our multidisciplinary expert panel developed stepwise guidance for emergency physicians approaching ED patients with HE. This ED-focused framework provides interventions for all patients with altered mental status and is accessible via the ACEP website and point-of-care app.

The ACEP has created and distributed point-of-care tools for nearly a decade, currently supporting 30 clinical conditions frequently encountered in the ED. These tools reach wide audiences through the. following: 1) free accessibility without paywalls (mobile application requires ACEP membership); and 2) easy-to-use formats (eg, high-yield checklists) leveraged at the bedside to facilitate data collection, orders, and result interpretation. As smartphones have become ubiquitous in EDs, handheld electronic clinical-decision support and just-in-time training are increasingly available and accepted.^31^ Artificial intelligence-enabled tools will likely further impact clinical decision support, with point-of-care content informing next-generation digital tools improving HE diagnosis and treatment.^32^

Nonspecific chief complaints, incomplete or inaccurate histories, and underlying liver disease necessitate broad differential diagnoses and extensive testing, increasing ED length of stay and evaluation complexity. The broad presentation range and rapid mental status fluctuations further complicate diagnosis. Patients may seek care for undiagnosed or undertreated HE consequences like infections, trauma, or medication errors. Emergency department pressures, including hallway care, inpatient boarding, and staffing shortages, challenge comprehensive exams and thorough histories, making collateral information needed for correct diagnosis difficult to obtain. These factors increase the risk of delayed or missed HE diagnosis. Despite these challenges, rapid identification and management of HE in the ED is essential, given the considerable mortality associated with altered mental status and high-grade HE among cirrhosis patients being cared for in this care setting.^34^

Looking ahead, further breakthroughs to reduce the prevalence of liver disease, along with tools to aid both the diagnosis and treatment of HE, are needed. Rapid, inexpensive diagnostic aids with high sensitivity and specificity, designed for use in the ED setting, would be a welcome addition to current testing approaches. Additionally, accessible treatments that rapidly correct HE, with regimens that maintain remission and achieve high compliance rates, would be ideal. The timing of treatment initiation is an important variable to optimize; for existing treatments with clear efficacy, studies are needed to demonstrate differences in patient outcomes between ED and inpatient care. For example, evidence supporting improved patient outcomes with rifaximin initiation within the typical timeframe of an ED visit (eg, within the first 6–8 hours of ED arrival) is needed.

## LIMITATIONS

Our approach had several limitations. First, we did not conduct a systematic literature review or report our findings in accordance with PRISMA standards.^35^ Our point-of-care tool should not be considered a clinical practice guideline; therefore, we did not apply the Appraisal of Guidelines Research and Evaluation (AGREE) reporting checklist.^36^ Additionally, this expert panel’s recommendations differ from those of ACEP clinical policies, which the ACEP Clinical Policies Committee develops through a separate process. Accordingly, grading the evidence and tying it to the strength of panel recommendations was out of scope for this project. Recommendations for HE grading and treatment were best supported by the literature, whereas other aspects of clinical care less likely to be explicitly studied, such as differential diagnosis and disposition destination, were consensus based.

Second, a common criticism of expert panel recommendations is that they are overrepresented by urban, academic physicians, which risks making them irrelevant to clinicians practicing in different settings. While we assembled a panel of U.S. clinicians from academic and community settings, as well as urban and rural settings, the panel selection process may not have resulted in panelists representative of the entire country, and it limits generalizability outside U.S. ED settings. This tool is not intended to represent a legal standard of care for emergency physicians. The ACEP recognizes the importance of the individual physician’s judgment and patient preferences. Lastly, several panelists reported financial relationships with entities relevant to HE, which may have introduced bias.

## CONCLUSION

The ACEP developed an evidence-based tool to evaluate and treat patients presenting to the ED with suspected or confirmed HE, with a focus on seamless integration into typical ED workflows. Emergency physicians should expect to encounter these patients more frequently over time as the population of patients with liver disease rises. Increased awareness of the spectrum of presentations, diagnostic criteria, and treatment options for HE is needed. Electronic tools such as the ACEP point-of-care apps are a valuable resource for rapid, accurate guidance. Further research is needed to answer key questions that could clarify the importance of timing specific treatments during the ED visit and aid clinicians in diagnosing HE more rapidly and accurately.

## Figures and Tables

**Figure 1:**
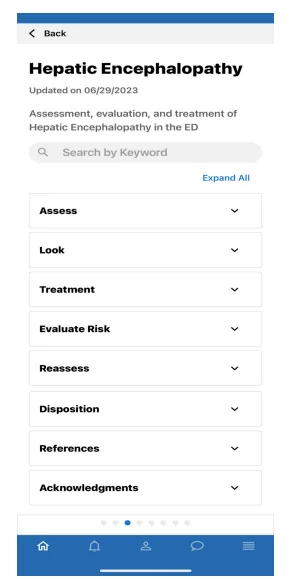
Screenshot of the American College of Emergency Physicians point-of-care tool for hepatic encephalopathy. Access the tool by opening the ACEP application, logging in with your ACEP credentials, selecting “Point-of-Care” in the upper left, and then choosing “Hepatic Encephalopathy” from the list.

**Figure 2:**
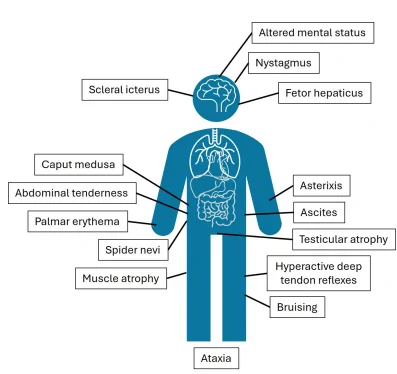
Signs and symptoms to look for during the physical examination in hepatic encephalopathy. Illustration of the physical manifestations of chronic liver disease found in the ACEP point-of-care tool for hepatic encephalopathy. *ACEP*, American College of Emergency Physicians.

**Figure 3:**
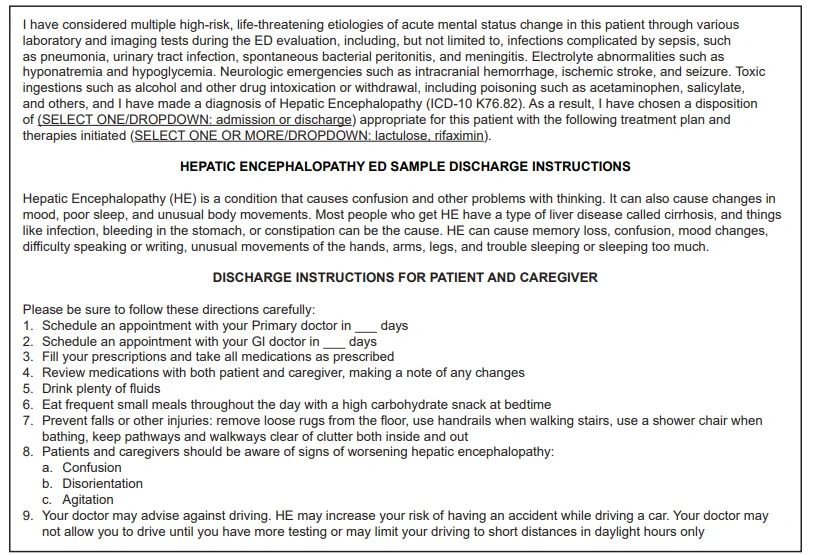
Sample dot phrase and emergency department discharge instructions for patients with hepatic encephalopathy. Example documentation exhibits to assist with the clinical note and discharge instructions provided to patients with hepatic encephalopathy in the emergency department, found in the ACEP point-of-care tool for hepatic encephalopathy. *ACEP*, American College of Emergency Physicians.

**Table 1:** The West Haven Criteria differentiate hepatic encephalopathy from subtle cognitive impairment to coma.^13^

Type of HE	Grade	Description
Covert	Minimal	No clinical evidence of mental changes Psychometric or neuropsychological testing may detect abnormalities
	1	Altered sleep rhythm Euphoria or anxiety Impairment of addition or subtraction Shortened attention span Trivial lack of awareness
Overt	2	Asterixis Disorientation for time Dyspraxia Inappropriate behavior Lethargy or apathy Obvious personality change
	3	Bizarre behavior Confused Gross disorientation Responsive to stimuli Somnolence to semistupor
	4	Coma

*HE*, hepatic encephalopathy.

**Table 2:** Laboratory testing recommended for patients with suspected hepatic encephalopathy.

Test	Comments
Ammonia level	Up to 40% of patients with HE may have a normal ammonia level
Blood alcohol level	Perform if indicated
BMP	Assess for metabolic disarray, especially hyponatremia, hypokalemia, hypoglycemia, and acute renal failure
CBC	Assess for evidence of associated infection, acute blood loss
Diagnostic paracentesis to evaluate for SBP	Up to 10% of patients with overt HE also have SBP
Drug levels Acetaminophen Digoxin Salicylates Valproic acid	Based on history or medication list
LFTs	Trend versus historical values, if available
Consider lumbar puncture	If meningitis is suspected without contraindications (eg, coagulopathy)
PT/INR	May be abnormal due to impaired hepatic synthetic function of coagulation factors
Radiology Consider right upper quadrant ultrasound (with Doppler) Consider noncontrast head CT	Assess for portal vein thrombosis Assess for intracranial hemorrhage
Toxicology screen	Perform if indicated*

Diagnostic testing considerations for the patient with chronic liver disease in the emergency department presenting with altered mental status found in the ACEP Point-of-Care tool for Hepatic Encephalopathy.

*ACEP*, American College of Emergency Physicians; *BMP*, basic metabolic panel; *CBC*, complete blood count; *CT*, computed tomography; *HE*, hepatic encephalopathy; *LFTs*, liver function tests; *PT/INR*, prothrombin time/International normalized ratio; *SBP*, spontaneous bacterial peritonitis.

* Urine drug screen is only warranted if the cause for presentation is unclear, and results will change emergency department treatment.

**Table 3:** Summary of hepatic encephalopathy treatment and disposition recommendations by patient diagnosis.

Patient Diagnosis	Disposition and Treatment Recommendation	Target Patient
Covert HE unrelated to the reason for visit Discharge home	Discharge home with PCP follow-up within 14 days If no PCP, use local processes for PCP and/or gastroenterology referral	WHC grade 1, no metabolic disarray or complications
Covert HE related to the reason for visit Discharge home	Discharge home with gastroenterology/hepatology specialist follow-up within 7 days, if available Consider discharge prescription for rifaximin 550mg BID If limited specialty clinic follow-up, consider gastroenterology consultation	WHC grade 1, no metabolic disarray or complications
Observation or inpatient hospitalization	Lactulose 30–45 mL (20–30 g) orally every 1–2 hours until mental status improves Consider rifaximin 550 mg BID to prevent recurrence Correct metabolic disturbances, CIWA for patients with alcohol use disorder ED observation protocol should include: - Explicit inclusion/exclusion criteria - Typical observation interventions - Endpoints for discharge with an outpatient follow-up plan versus inpatient admission Recommend gastroenterology consult, if available At discharge, recommend follow-up with gastroenterology/hepatology clinic within 3–5 days, if available	WHC grade 1 with barriers to follow-up, or WHC grade 2 with near-normal mental status and/or mild metabolic disarray or complications with anticipated length of stay less than two midnight s
Overt HE Inpatient hospitalization	Administer lactulose 30–45 mL (20–30 g) orally or through nasogastric tube^†^ every 1–2 hours until mental status improvement Consider adding rifaximin 550 mg BID to prevent recurrence Recommend gastroenterology consult, if available	WHC grade 2–3, arousable and redirectable, minimal or moderate metabolic disarray or complications
Overt HE ICU hospitalization	If the patient is intubated, administer lactulose 30–45 mL (20–30 g) via nasogastric tube every 1–2 hours until mental status improvement If not intubated, administer lactulose 200 g rectally hourly until improvement in mental status PEG-3350 4 L can be administered via nasogastric tube as adjunctive therapy Consider adding rifaximin 550 mg BID once the patient can take medications orally to prevent recurrence Recommend gastroenterology consult, if available	WHC grade 4, obtundation, and severe metabolic disarray or complication

Treatment and disposition recommendations for the emergency department patient with hepatic encephalopathy according to the degree of illness found in the ACEP point-of-care tool for hepatic encephalopathy

*ACEP*, American College of Emergency Physicians; *CIWA*, Clinical Institute Withdrawal Assessment for Alcohol; *BID*, twice daily; *HE*, hepatic encephalopathy; *PCP*, primary care physician; *WHC*, West Haven criteria.
